# Isolation and genetic characterization of a novel 2.2.1.2a H5N1 virus from a vaccinated meat-turkeys flock in Egypt

**DOI:** 10.1186/s12985-017-0697-5

**Published:** 2017-03-09

**Authors:** Ahmed H. Salaheldin, Jutta Veits, Hatem S. Abd El-Hamid, Timm C. Harder, Davud Devrishov, Thomas C. Mettenleiter, Hafez M. Hafez, Elsayed M. Abdelwhab

**Affiliations:** 1 Institute of Poultry Diseases, Free University of Berlin, Königsweg 63, 14163 Berlin, Germany; 2 Friedrich-Loeffler-Institut, Federal Research Institute for Animal Health, Suedufer 10, 17493 Insel Riems-Greifswald, Germany; 3 Department of Poultry Diseases, Faculty of Veterinary Medicine, Alexandria University, Alexandria, Egypt; 4 Faculty of Veterinary Medicine, Damanhur University, Damanhur, Egypt; 5 Moscow State Academy of Veterinary Medicine and Biotechnology, Moscow, Russia

**Keywords:** Vaccinated turkeys, H5N1, Vaccination failure, Clade 2.2.1.2, Avian influenza, Egypt

## Abstract

**Background:**

Vaccination of poultry to control highly pathogenic avian influenza virus (HPAIV) H5N1 is used in several countries. HPAIV H5N1 of clade 2.2.1 which is endemic in Egypt has diversified into two genetic clades. Clade 2.2.1.1 represents antigenic drift variants in vaccinated commercial poultry while clade 2.2.1.2 variants are detected in humans and backyard poultry. Little is known about H5N1 infection in vaccinated turkeys under field conditions.

**Case presentation:**

Here, we describe an HPAI H5N1 outbreak in a vaccinated meat-turkey flock in Egypt. Birds were vaccinated with inactivated H5N2 and H5N1 vaccines at 8 and 34 days of age, respectively. At 72^nd^ day of age (38 days post last vaccination), turkeys exhibited mild respiratory signs, cyanosis of snood and severe congestion of the internal organs. Survivors had a reduction in feed consumption and body gain. A mortality of ~29% cumulated within 10 days after the onset of clinical signs. Laboratory diagnosis using RT-qPCRs revealed presence of H5N1 but was negative for H7 and H9 subtypes. A substantial antigenic drift against different serum samples from clade 2.2.1.1 and clade 2.3.4.4 was observed. Based on full genome sequence analysis the virus belonged to clade 2.2.1.2 but clustered with recent H5N1 viruses from 2015 in poultry in Israel, Gaza and Egypt in a novel subclade designated here 2.2.1.2a which is distinct from 2014/2015 2.2.1.2 viruses. These viruses possess 2.2.1.2 clade-specific genetic signatures and also mutations in the HA similar to those in clade 2.2.1.1 that enabled evasion from humoral immune response. Taken together, this manuscript describes a recent HPAI H5N1 outbreak in vaccinated meat-turkeys in Egypt after infection with a virus representing novel distinct 2.2.1.2a subclade.

**Conclusions:**

Infection with HPAIV H5N1 in commercial turkeys resulted in significant morbidity and mortality despite of vaccination using H5 vaccines. The isolated virus showed antigenic drift and clustered in a novel cluster designated here 2.2.1.2a related to viruses in poultry in Israel, Gaza and Egypt. Enforcement of biosecurity and constant update of vaccine virus strains may be helpful to protect vaccinated birds and prevent spillover infection to neighbouring countries.

**Electronic supplementary material:**

The online version of this article (doi:10.1186/s12985-017-0697-5) contains supplementary material, which is available to authorized users.

## Background

### Findings

Avian influenza (AI) viruses are members of the genus influenza virus A in the family *Orthomyxoviridae* and possess a single-stranded RNA genome composed of eight gene segments encode at least ten viral proteins [1]. The two envelope glycoproteins hemagglutinin (HA) and neuraminidase (NA) are responsible for virus attachment to and release from the host cell, respectively. HA is the main determinant of virulence and immunogenicity. The polymerase basic (PB) 1 and 2, polymerase acidic (PA) and nucleoprotein (NP) represent the minimal replicative unit of the virus. In addition, the bicistronic matrix (M) and non-structural (NS) gene segments each encode two proteins: M1 and M2, and NS1 and nuclear export protein (NEP), respectively. Some AI viruses possess PA-x and PB1-F2 proteins encoded by a frameshift of PA and PB1 gene segments. All proteins are associated with the virion except the nonstructural NS1 and PB1-F2 [1]. To date, 16 HA (H1-16) and 9 NA (N1–N9) subtypes have been detected in wild birds, the natural reservoir of the virus. Transmission of AI viruses from wild birds to domestic poultry resulted in severe losses in the poultry industry and endangered human health. In contrast to the low pathogenic (LP) nature of AI in wild birds, in domestic poultry H5 and H7 viruses may shift to high pathogenic (HP) forms after the acquisition of point mutations or swapping of gene segments between different AI subtypes [2]. In 1996/1997, an HPAI H5N1 virus was generated by the reassortment of the HA and NA segments of H5N1 with internal segments from H6N1 or H9N2 viruses in live bird markets in Hong Kong [3]. Since then, the virus has evolved into 10 different phylogenetic clades (designated clade 0 to 9) and tens of subclades according to the diversity of the HA gene [4].

Turkeys play an important role in the adaptation of wild-bird viruses to gallinaceous poultry. They also act as mixing vessel for the generation of reassortant viruses sharing signatures of human, avian and swine influenza viruses [5–8]. The susceptibility of turkeys to LP and HP AI viruses is higher than that of chickens and waterfowl [9, 10]. Severe mortality has been reported in natural outbreaks due to infection with HPAIV H7N1 [9], and recently with H5N2 and H5N8 in North America [11, 12] and Germany [13]. Therefore, vaccination of turkeys with a variety of H5 virus vaccines was implemented in the field to protect turkeys from lethal infection by HPAIV. The frequency and time of vaccination as well as the dose of the inactivated vaccine in turkeys are mostly different from these of chickens [14]. Antigenic-drift variants represented a serious challenge for the efficacy of current vaccines in poultry. Field reports on vaccinal breaks in chicken flocks are common [15–19], whereas little is known about H5N1 in turkeys [20].

Egypt is one of the few countries with an endemic status of clade 2.2.1 HPAI H5N1 virus, which diversified into two distinct subclades designated 2.2.1.1 and 2.2.1.2. Viruses in clade 2.2.1.1 circulated in vaccinated poultry, including turkeys, from 2007 to 2014 despite intensive blanket vaccination using over 20 diverse H5 vaccines [21–23]. Meanwhile, clade 2.2.1.2 viruses were observed mainly in backyard birds and humans, and recently in the commercial birds. They caused severe socioeconomic losses in the poultry industry and posed a serious pandemic threat because of their affinity to human-type receptors [24, 25]. The emergence of antigenic-drift variants, improper vaccination, and or immunosuppression are commonly held responsible for vaccination failure mainly in chickens. However, no detailed data are available on turkeys [21].

In this manuscript, we described a recent outbreak with HPAI H5N1 in a vaccinated meat turkey flock at 10 weeks of age. The flock was vaccinated twice with H5 vaccines at 8 and 34 day of age. Details on feed consumption, body weight and mortality are provided. Full genome characterization of the isolated virus in relation to the vaccine and circulating viruses were analyzed.

## Case Presentation

### Clinical examination

**Fig. 1:**
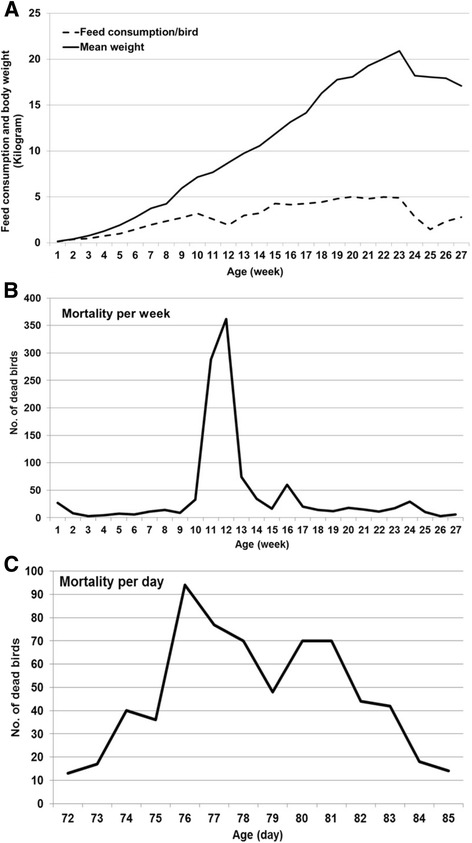
Performance (**a**) and number of dead birds of the meat-turkey flock in this study per week (**b**) or per day since the appearance of clinical signs (**c**)

Data on the feed consumption and mean body weight per week was calculated for living birds and summarized in Fig. 1. Generally, the feed consumption was normal and increased with time. Likewise, birds had a steady increase in body weight. At weeks 10 to 12 a remarkable decrease in feed consumption and body weight was observed (Fig. 1a), when birds started exhibiting clinical signs of mild respiratory distress, sneezing and coughing accompanied with sudden onset of mortalities. High mortality started at week 10 and increased in weeks 11 and 12 then decreased at weeks 13 and 14, where 33, 288, 362, 74 and 34 birds died, respectively. A second shorter increase of mortality at week 16 was observed (Fig. 1b). The highest number of birds died between 74 and 83 days of age (Fig. 1c). Total mortality amounted to 37.1% while 29% of turkeys died within 10 days following onset of disease in the holding. Since birds had been vaccinated twice and there was no recent record of HPAIV H5N1 in that region, postmortem examination was conducted on the farm. Birds suffered severe congestion of all internal organs including lungs. The preliminary early diagnosis suggested a bacterial infection and therefore antibiotics were used to treat the flock, but without success. Emergency vaccination with live NDV vaccine was also administered via drinking water at day 73 (Table 1) due to the low anti-NDV HI titer (GMT = 2^4^ - 2^5^; data not shown).

**Table 1:** Vaccination program of the flock

Vaccine	Type	Age of vaccination (Day)	Route of Vaccination
NDV (Hitchner B1) + IB	Live	6	Ocular
NDV + AI (H5N2; Mexico/94)	Inactivated	8	Subcutaneous
Avian Metapneumovirus (TRT)	Live	15	Ocular
NDV (Clone 30)	Live	20	Ocular
HE	Live	26	Drinking Water
AI (H5N1; Re-5)	Inactivated	34	Subcutaneous
Poxvirus	Live	40	Wing Web
*P. multocida*	Inactivated	41	Subcutaneous
NDV (Clone 30)	Live	50	Drinking Water
NDV (Lasota)	Live	73	Drinking Water

*ND* = Newcastle Disease, *IB*=, Infectious Bronchitis, *AI* = Avian Influenza, *TRT* = Turkey rhinotracheitis, *HE* = Hemorrhagic Enteritis

H5N2 vaccine is seeded by A/chicken/Mexico/232/CPA/94(H5N2)

Re-5 vaccine strain carry the HA and NA genes of A/duck/Anhui/1/2006(H5N1)

### Laboratory diagnosis

Swab samples were inoculated into 9-day-old SPF ECE and examined by HA/HI tests and RT-PCR. All embryonated eggs died within 48 h post inoculation and HA test was positive from allantoic fluid. Avian influenza virus was confirmed using HI test. Samples were submitted to the FLI for further confirmation. The results of RT-qPCRs confirmed the presence of H5N1 and revealed negative results for H7, H9 and N2. The cross reactivity of TK16 was studied by HI test using serum samples generated against different H5 viruses as shown in Table 2. HI titers produced by TK16 were 4 – 8 fold lower than these produced by the homologous viruses in clades 2.2.1.2, 2.2 and clade 1 and 32 fold lower than those in clades 2.2.1.1 and 2.3.4.4 (Table 2). This difference in antigenic drift was not correlated with the global HA gene or protein identity ratios (Table 2).

**Table 2:** Antigenic characterisation of A/turkey/Egypt/AR1507/2016(H5N1) (TK16) isolated from vaccinated turkeys in this study

Serum samples against:	Clade	HI titer (log_2_) against		HA identity*
		TK16	Homologous antigen	
A/chicken/Egypt/AR234-FAOF8NLQP/2014 (H5N1)	2.2.1.2	7	9	99.4/100
A/chicken/Egypt/0879-NLQP/2008 (H5N1)	2.2.1.1	5	10	94.7/94.2
A/teal/Germany/WV632/2005 (H5N1)	2.2	6	9	88.7/90.6
A/turkey/Germany-MV/R2472/2014 (H5N8)	2.3.4.4	2	7	88.7/90.6
A/Vietnam/1194/2004 (H5N1)	1	6	8	93.2/95.1

HA identity ratio was calculated for nucleotides and deduced amino acids after alignment of the gene sequence and deduced amino acids using Bio Edit by the equation

100 - (Length of gene segment - Number of mutations/Length of gene segment)

### Sequence analysis

**Fig. 2:**
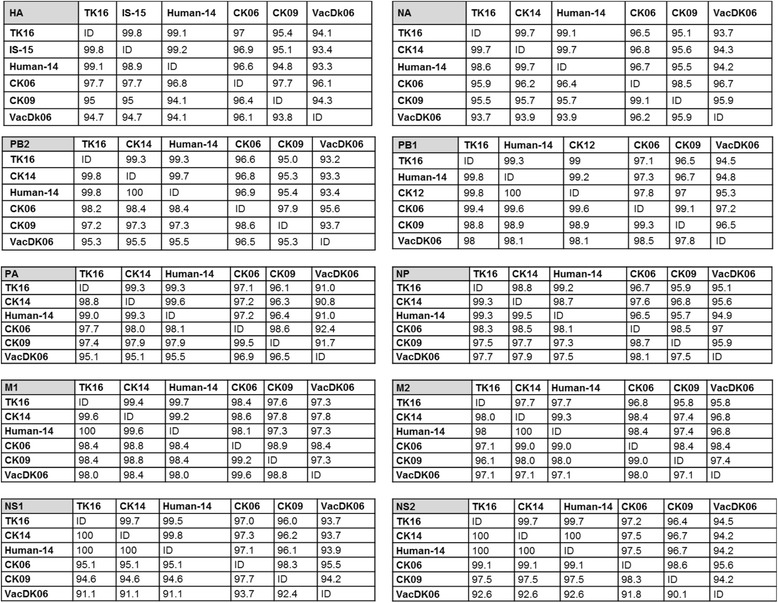
Identity matrix of all gene segments of A/turkey/Egypt/AR1507/2016 to relevant H5N1 viruses. Nucleotide and amino acids identity matrices of all proteins of A/turkey/Egypt/AR1507/2016 compared to related viruses of chicken and human origins, the ancestral 2006 virus, the variant 2.2.1.1 strain and vaccine strain. TK16 = A/turkey/Egypt/AR1507/2016, IS-15 = A/turkey/Israel/633/2015 (clade 2.2.1.2), Human-14 = A/Egypt/MOH-NRC-7271/2014 (clade 2.2.1.2), CK06 = A/chicken/Egypt/06553-NLQP/2006 (clade 2.2.1), CK14 = A/chicken/Egypt/A10351A/2014 (clade 2.2.1.2), CK09 = A/chicken/Egypt/096 L-NLQP/2009 (clade 2.2.1.1), and VacDk06 = vaccine strain A/duck/Anhui/1/2006 (clade 2.3.4); ID = identical, Identity of the nucleotides and amino acids was calculated by the equation: 100 - (Length of gene segment - Number of mutations/Length of gene segment)

Nucleotide and amino acid (aa) identities for each gene were calculated by BioEdit and manually and summarized in Fig. 2, a list of mutations is supplied in Additional file 1: Table S1.

### Hemagglutinin (HA) gene

**Fig. 3:**
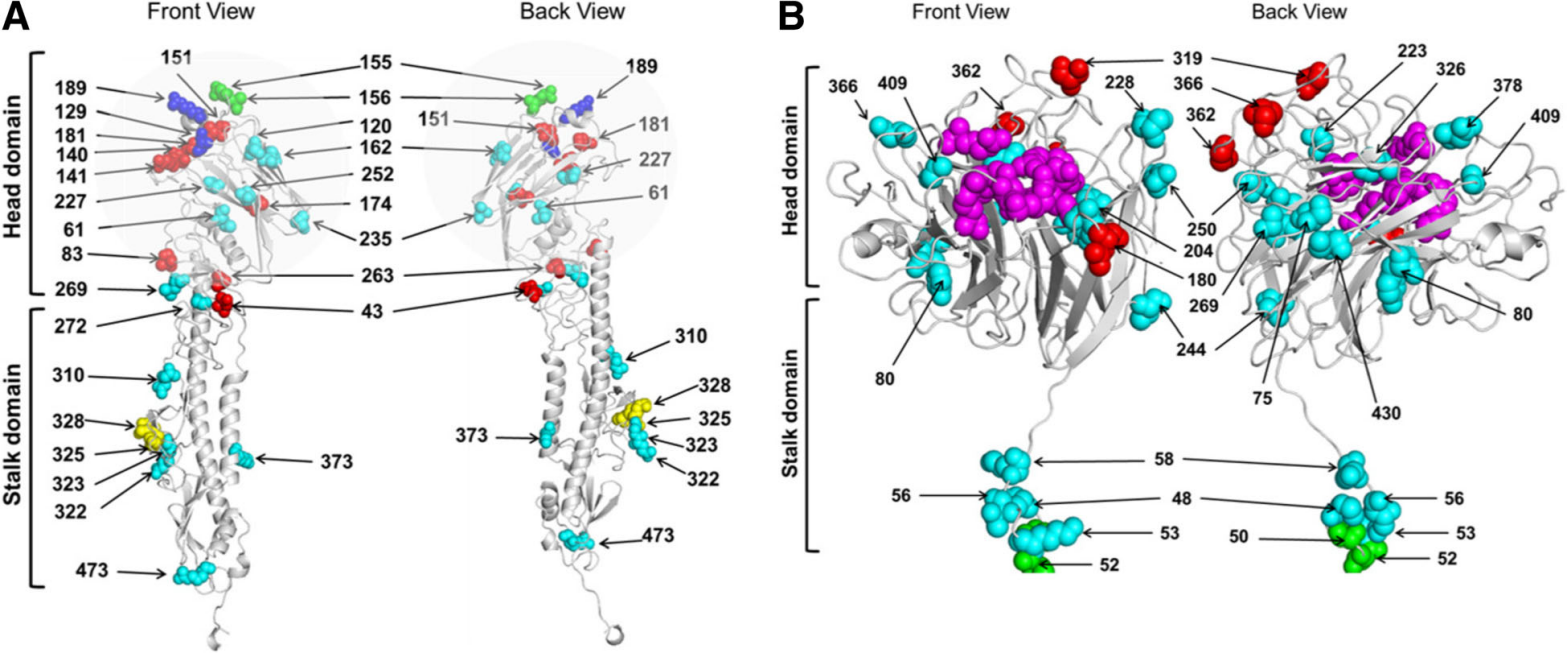
Predicted location of amino acids in the HA and NA proteins of A/turkey/Egypt/AR1507/2016(H5N1). Shown are mutations in HA (**a**) and NA (**b**) in TK16 compared to the vaccine strain Dk2006. (**a**) Most of mutations in the HA occurred in the head domain (*transparent grey circle*): mutations in the immunogenic epitopes (*red*), receptor binding sites (*blue*), de-glycosylated sites (*green*), in the proteolytic cleavage site (*yellow*) and those with unknown function (*cyan*). (**b**) in the NA, mutations occurred in the stalk (*n* = 11; while residues 20, 29, 34, 44 and 46 are not shown in the 3D structure) and head domain (*n* = 17), the sialidase functional and framework sites (*magenta*), mutations in the immunogenic epitopes (*red*), glycosylated sites (*green*), and those with unknown function (*cyan*) are shown. All mutations are imposed on the HA and NA of the parent A/chicken/Egypt/NLQP-06207/2006 virus

The HA of TK16 shared 95.1% aa identity with the vaccine VacDK06 strain with 27 aa differences: one aa in the signal peptide, 24 aa in the HA1 and 2 aa in the HA2. These mutations are in residues 43, 61, 83, 120, 129, 140, 141, 151, 155, 156, 162, 174, 181, 189, 227, 235, 252, 263, 269, 272, 310, 322, 323, 325, and 328 in the HA1 subunit, in addition to 373 and 473 in the HA2 subunit. Substitutions in residues 155 and 156 resulted in the loss of a potential glycosylation site in the head domain of TK16 (Fig. 3). The only available sequence of Mexican H5N2 virus is the HA1 which shared 83.5% aa and 78.4% nucleotide identities with the current Tk2016. Compared to viruses from 2006, the mature HA possessed 46 nucleotide differences, 14 out of them resulted in 11 aa changes, whereas 32 were silent. The amino acid differences are in positions 43, 120, 129, 151, 154, 155, 162, 272, 325, 373, and 537 (Fig. 3). Compared to the recent viruses in clade 2.2.1.2 from human and poultry, there was no new amino acid change but three non-synonymous mutations.

### Neuraminidase (NA)

The NA of the virus shared 93.8% aa identity with the vaccine strain with 28 aa substitutions in residues 20, 29, 34, 44, 46, 48, 50, 52, 53, 56, 58, 75, 80, 180, 204, 221, 228, 244, 250, 269, 284, 319, 326, 362, 366, 378, 409 and 430 (Fig. 3). The NA active and framework sites as well as the hemadsorption sites are conserved. TK16 possesses markers sensitive to the neuraminidase inhibitor Oseltamivir: V96, I97, E99, K130, D179, I203, Y233 and H255 (equivalent to N2 numbering residues 116, 117, 119, 150, 197, 222, 252 and 274, respectively). Compared to viruses from 2006 there were 16 aa differences in residues 20, 29, 34, 46, 48, 56, 74, 91, 180, 204, 221, 244, 284, 319, 378, and 430. Only 3 new mutations were found: N180S, V221I and D378E. Additionally, mutations in position 50–52 resulted in a potential GS which was not present in the parent 2006-virus. Compared to the recent viruses in clade 2.2.1.2b, they are only different in position 46; the 2.2.1.2a possesses aspartic acid while 2.2.1.2b possesses Asparagine in addition to one-non-synonymous mutation.

### Other gene segments

Identity matrices of all gene segments indicated that TK16 has ≥98.8 nucleotide and aa similarity to recent poultry and human isolates in Egypt, Israel and Gaza, and up to 91% identity to the vaccine strain (Fig. 2). Compared to the parental 2006-virus and variant 2.2.1.1 clade TK16 shared ≥96.7 and ≥95 nucleotide and ≥95.1 and ≥94.6 aa identities, respectively. Mutations in TK16 compared to other viruses in Egypt and the vaccine strain are summarized in Additional file 1: Table S1. Most of mutations were similar to previous reports [25–27] except in PB2 (*n* = 7), PB1 (*n* = 1), PA (*n* = 11), NP (*n* = 4), M1 (*n* = 1), M2 (*n* = 2) and NS1 (*n* = 9) as shown Additional file 1: Table S1.

### Phylogenetic analysis

**Fig. 4:**
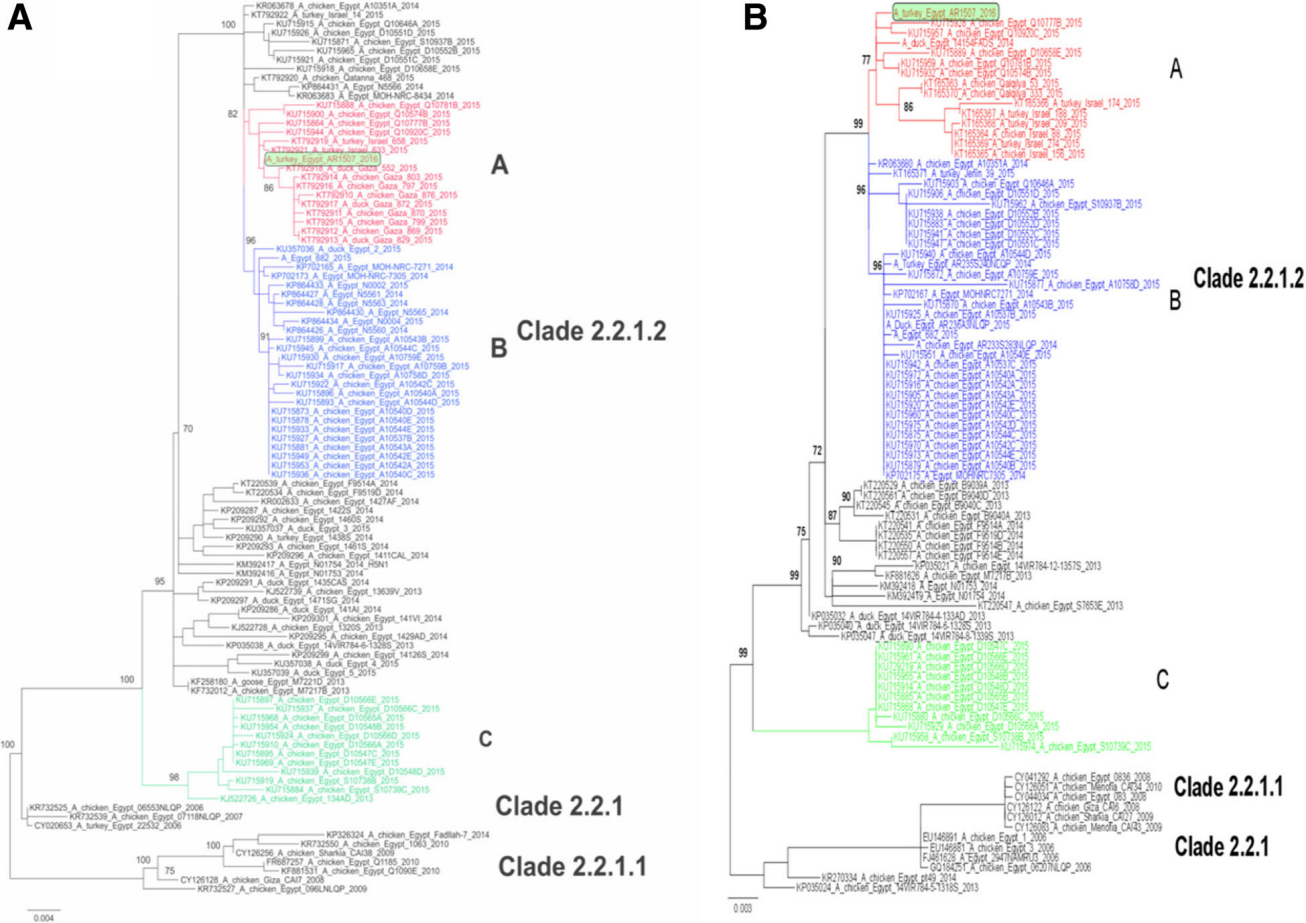
Phylogenetic analysis of the HA (**a**) and NA (**b**) gene segments of A/turkey/Egypt/AR1507/2016 to other related H5N1 viruses in poultry and humans. Phylogenetic analysis of the HA (**a**) and NA (**b**) genes of Egyptian viruses showing different genetic clades. Viruses in 2015 from Egypt, Gaza and Israel are clustered in clade 2.2.1.2 in three distinct subclades denoted A, B and C. A/turkey/Egypt/AR1507/2016 is located in subclade A along with recent viruses isolated in 2014 and 2015 in poultry in Egypt, Gaza and Israel. Subclade B represents viruses of human and poultry origin in 2014/2015, and subclade C contains viruses from poultry origin in Egypt in 2015

Phylogenetic relatedness of all gene segments was determined by Bayesian inference analysis in MrBayes using the best fit model predicted by jModelTest. Phylogenies of all gene segments of TK16 showed similar topology. In the HA, the recent viruses in clade 2.2.1.2 grouped in three distinct subclade designated subclade A, B and C. TK16 belonged to clade 2.2.1.2a clustering with gene sequences from recent viruses isolated in 2014 and 2015 in poultry in Egypt, Gaza and Israel (Fig. 4). This new cluster is supported by a high posterior probability value and possesses 3 unique non-synonymous mutations compared to viruses in group 2.2.1.2b which were isolated in the recent upsurge in 2014/2015. Likewise, the NA was also very closely related to the available sequences of viruses isolated in poultry in Israel and Gaza forming along with viruses from poultry in 2014/2015 in Egypt a distinct cluster from human viruses in 2014/2015. Similar topology was observed for the phylogenies of other gene segments (Additional file 2 Figure S1).

## Discussion

Vaccination of domestic poultry with H5 vaccines has been implemented in several countries to limit the spread of H5N1 AI virus. Many studies have shown that vaccination of poultry with antigenically related vaccines protected birds from morbidity, mortality and virus excretion [28–32]. However, insufficient efficacy of the vaccines was reported after the infection with antigenic drift variants [16, 18, 19]. A recent study has also shown that H5N1 clade 7.2 viruses have not been eradicated from poultry in China through intensive vaccination [33]. In the contrast to chickens, less is known about HPAI H5N1 infection in vaccinated turkeys. In this manuscript, we describe a vaccinal break in a meat-turkey flock vaccinated twice with two different inactivated H5 vaccines. Clinical signs and sudden onset of mortality were reported few weeks after the last vaccination. Interestingly, 29% mortality within 10 days was observed and surviving turkeys showed a transient drop in feed consumption and body weight. In the available literature no data are available on the efficacy of Re-5 to protect turkeys against challenge with Egyptian H5N1-like viruses. Re-5 was efficient to protect chickens from mortality after experimental infection with early H5N1 viruses in clade 2.2.1 and 2.2.1.1, although virus shedding was significantly higher than in birds vaccinated with a local vaccine [34]. In an experiment, 20 to 100% mortality during a 10 days observation period were described in turkeys vaccinated with either H5N1 or H5N2 vaccines after challenge with Egyptian HPAIV H5N1 of clade 2.2.1.1 [35].

In this study, data about the levels of anti-H5 antibodies in serum samples from the infected turkey flock are unfortunately missing. However, antigenic characterisation indicated antigenic drift particularly against serum samples from clade 2.2.1.1 in poultry in Egypt and to lesser extent against serum samples from genetically related 2.2.1.2 virus (Table 2). Also, TK16 showed remarkable antigenic drift against the recent H5N8 2.3.4.4 viruses, descendent clade from the vaccine strain. Unlike 2.2.1.1 viruses which circulated in Egypt from 2007 to 2014, viruses in clade 2.2.1.2 showed less, if any, antigenic variation, and vaccination of chickens was efficient even with highly diverse H5 vaccines derived from the Egyptian viruses [34, 36]. However, TK16 showed a substantial genetic diversity at the protein level when compared with VacDK06 (clade 2.3.4) especially in HA and NA. In HA TK16 possessed up to 28 amino acids different from VacDK06. In addition to the 2.2.1.2 clade-specific signature (residues D43N, S120D, ∆129S I151T, R162K, G272S, R325K) which enabled efficient replication of the Egyptian 2.2.1.2 viruses in human cells and mice [24, 37, 38], several mutations in the immunogenic epitopes (L71I, I83A, R140K, S141P, D155N, A156T, V174I, P181S, T263A) [39] mostly in the HA1 head domain (Fig. 3) were observed in the new 2.2.1.2a subclade including TK2016. Some of these mutations are shared with viruses in clade 2.2.1.1 (e.g. L71I, R140K, S141P and A156T). These enabled the latter viruses to escape from the humoral immune response induced by H5 vaccines [40] and resulted in vaccination failure in chickens [34]. In the NA, unlike 2.2.1.1 clade viruses which contain only a single amino acid change compared to the viruses in 2006 and few mutations compared to the vaccine strains [41], clade 2.2.1.2 viruses including TK16 possess several substitutions in the NA. Residue A20, I29, I34, S44, A46, S48, S50, T52, K53, A56, and K58 are located in the stalk region [42] while the others are positioned at the head domain of the NA monomer (Fig. 3). Some amino acid changes are in the NA immunogenic epitope A (362, 366 equivalent to N2 numbering 385, 389), epitope B (180 equivalent to N2 199) or epitope C (residue 319 equivalent to 339 N2 numbering) [43]. Meanwhile, 378 (equivalent to 402 N2 numbering) is located close to immunogenic epitope A, 204 (N2 numbering 223) close to epitope B and between two framework and functional residues of the sialidase, and 326 (equivalent to 349 N2 numbering) is close to epitope C [43]. Together, continuous circulation of clade 2.2.1.2 viruses in vaccinated poultry resulted in the emergence of another antigenic variant sub-clade distinct from the human viruses in clade 2.2.1.2 resembling the situation in 2006 when the parental clade 2.2.1 diverged into two distinct clades: 2.2.1.1 in poultry in 2007–2014 and 2.2.1.2 in non-vaccinated poultry and humans in 2008–2016.

The source of infection in this flock is not yet clear. There was no infection in adjacent layers-flock premises according to the official reports. Since the turkey flock was vaccinated against NDV several days before starting the increased mortality the vaccination crew may be responsible for the introduction of the virus. This remains speculative, however. Moreover, phylogenetic analysis indicated a very close relationship of TK16 with recent viruses from Israel and Gaza in 2015. Since the flock was kept in an open system house, transmission by feral birds (e.g. pigeons, sparrows or egrets) or migratory birds (e.g. waterfowl) can not be excluded. Legal poultry trade between Egypt and both regions is not known and smuggling of poultry or feed is unlikely.

## Conclusions

Our study is important because so far no data on the infection of vaccinated turkeys with HPAI H5N1 virus under field conditions are available. Likewise, data are scarce about performance (e.g. feed consumption and body weight) of turkeys during the course of HPAI H5N1 infection. Antigenic characterisation indicated genetic drift. Full genome analysis along with other viruses from Egypt, Israel and Gaza indicated the emergence of a new cluster supported by high posterior probability and therefore designated clade 2.2.1.2a. Further research is required to evaluate the efficiency of current vaccines to protect turkeys against infection with the new viruses. Also, mutations demonstrated here with unknown biological functions should be further investigated. The new cluster carries signatures from both chicken-adapted and human-adapted clades which warrants vigilance to prevent at an early stage the spread of human-adapted strains in Egypt.

### Methodology

#### Flock history

The affected population was a meat-turkey flock containing 2040 male BUT-Big6 turkeys. They were reared in an open system house with good hygienic conditions. At a distance of about three-hundred meters a layer chicken farm was located. The feed was manufactured in the company feed mill supplemented by anticoccidials, anti-microbial growth promotors and antimycotoxins; all ingredients were used according to the species requirement. Water supply was from an underground source. Birds were vaccinated subcutaneously at 8 day-of age with a bivalent vaccine containing A/chicken/Mexico/CPA/1994(H5N2) and Newcastle disease virus (NDV), and boostered for AI at 34 days old using Re-5 vaccine containing HA and NA from A/duck/Anhui/1/2006(H5N1) clade 2.3.4 (designated VacDK06) and other gene segments from A/Puerto Rico/1/1934(H1N1). Additionally, the flock was revaccinated and/or vaccinated against NDV, Avian Metapneumovirus (Turkey rhinotracheitis-TRT), Hemorrhagic enteritis, poxvirus, and *Pasteurella multocida* (Table 1).

#### Sample collection

Cloacal and oropharyngeal swabs were collected from 25 birds at the onset of the clinical signs, or from dead birds. Swabs were placed in transport medium, consisting of phosphate-buffered saline containing glycerol, penicillin (2000 U/ml), gentamicin (250 mg/ml) and nystatin (500 U/ml). Collected swab samples were sent to the Faculty of Veterinary Medicine, Damanhur University, Egypt for routine diagnosis. Samples were also sent to the OIE and National Reference Laboratory for Avian Influenza, Federal Research Institute for Animal Health, Friedrich-Loeffler-Institut, Germany for further virus characterization. In this study, no experimental research was conducted and all turkeys were handled according to the standard guidelines.

#### Virus isolation

Virus isolation from swab samples was done in 9-day-old specific-pathogen-free (SPF) embryonated chicken eggs (ECE) according to the OIE guidelines [44]. Inoculated eggs were examined daily for 3 – 5 days. Eggs with dead embryos were kept at 4 °C for 24 h. Allantoic fluid was collected and examined using the standard hemagglutination test and 1% chicken erythrocytes. Allantoic fluids of positive eggs were pooled together and tested by the hemagglutination inhibition (HI) test for the presence of H5 and ND viruses using specific antisera [44].

#### Antigenic characterisation

Sera against different H5 viruses as well as homologous antigens were obtained from the repository of the FLI. Cross-reactivity of TK16 against different sera was studied by hemagglutination inhibition (HI) test. The HI test was done against 1% chicken erythrocytes and 4 HA units of each antigen in 96-well plastic V-bottomed microtiter plates according to the OIE manual [44]. The test was done in duplicates and the titers produced against TK16 were compared to those produced by the homologous antigens.

#### Real-time reverse-transcription polymerase chain reaction (RT-qPCR)

Viral RNA was extracted from swab media using the QIAamp Viral RNA Kit (Qiagen, Hilden, Germany) following the manufacturer’s instructions. RT-qPCR specific for the AIV M gene was followed by RT-qPCRs specific for the HA of H5, H7, H9 HA-subtypes and N1 and N2 NA-subtypes using primers published by Hoffmann et al. [45].

#### Sequencing and sequence analysis

Complementary cDNA was generated from 4 μl RNA using Omniscript RT Kit (Qiagen, Hilden, Germany) along with a primer specific to the conserved 12 nucleotide of 3’end of the viral RNA as previously published [46]. The HA, NP, NA, M and NS gene segments were amplified using universal primers and 1 μl of cDNA according to Kreibich et al. [47], whereas the PB2, PB1 and PA genes were amplified using internal primers to generate long overlapping regions (primers are available upon request). All PCR reactions were performed in Thermocycler machine (Eppendorf, Hamburg, Germany) as previously done [47]: an initial denaturation step (98 °C 30 s), followed by 35 cycles each consisting of 98 °C 10s, 60 °C 30s, 72 °C 6 min and final elongation (72 °C 5 min) utilizing 2 U Phusion High-Fidelity DNA Polymerase (New England BioLabs, Frankfurt am Main, Germany) according to the manufacturer’s guidelines. Fragments’ sizes were determined by electrophoresis in 1% agar gel in comparison to the GeneRuler™ DNA ladder (Thermo Scientific, Germany). Amplicons were excised and purified using the QIAquick Gel Extraction Kit (Qiagen, Hilden, Germany). The purified PCR products were sequenced using a BigDye Terminator v1.1 Cycle Sequencing Kit (Applied Biosystems, Langen, Germany) and appropriate primers purified with NucleoSEQ® Columns (MACHEREY-NAGEL GmbH & Co. KG). Sanger sequencing was conducted in a 3130 Genetic Analyzer (Applied Biosystems). Sequences of full genome of the virus isolated in this study were submitted to the Global Initiative on Sharing All Influenza Data (GISAID) and assigned accession numbers: EPI827065 to EPI827072. N-linked glycosylation in the HA and NA proteins were predicted by NetNGlyc 1.0 Server. The location of mutations described in the HA and NA in this study was predicted on the tertiary structure of the corresponding proteins of the parent 2.2.1 virus (A/chicken/Egypt/NLQP-06702/2006) using SWISS MODEL (http://swissmodel.expasy.org/) and then viewed and edited by geneious software suite v.8.1.3 (Biomatters, Auckland, New Zealand).

#### Phylogenetic analysis

Sequence similarity to each gene segment of the virus isolated in this study was detected by Basic Local Alignment Search Tool (BLAST) database available at the NCBI [48]. Sequences with the maximum BLAST scores and identity percentages were selected. Moreover, all viruses in 2014–2015 from Egypt, Israel and Gaza as well as representative viruses from clades 2.2.1 and 2.2.1.1 were downloaded. All sequences were aligned by Multiple Alignment using Fast Fourier Transform (MAFFT) [49] and further viewed and edited by BioEdit 7.1.7 [50]. Amino acid sequences were deduced from gene sequences and identity matrices were calculated using BioEdit and manually. Bayesian inference phylogenetic trees for each gene segment were generated using MrBayes 3.2.6 [51] under best-fit models calculated by jModelTest [52]. Two parallel runs consisted of four chains of Markov Chain Monte Carlo (MCMC) iterations for 10^8^ generations were selected for each nucleotide sequence. For each phylogenetic tree A/goose/Guangdong/1/1996(H5N1) (Clade 0) was specified as the out-group. Graphic outputs were produced by FigTree (http://tree.bio.ed.ac.uk/software/figtree/) and Inkscape 0.91 (www.inkscape.org).

## Additional files

Additional file 1: Table S1. Amino acids differences from A/turkey/Egypt/AR1507/2016 and ancestral 2006 virus, vaccine strain and closest human isolate (DOC 36 kb)
Additional file 2: Figure S1. Phylogenetic analysis of internal protein coding gene segments of A/turkey/Egypt/AR1507/2016 to other related H5N1 viruses in poultry and humans. Phylogenetic analysis of non HA/NA genes of Egyptian viruses showing different genetic clades. Viruses in 2015 from Egypt including A/turkey/Egypt/AR1507/2016 are clustered in clade 2.2.1.2. The tree was generated by MrBayes under the best-fit model selected by jModelTest. (PDF 701 kb)

## Abbreviations

aa: Amino acids
AIV: Avian influenza virus
BLAST: Basic Local Alignment Search Tool
ECE: Embryonated chicken eggs
GISAID: Global Initiative on Sharing All Influenza Data
GMT: Geometric mean titer
GS: Glycosylation site
HA test: Hemagglutination test
HA: Hemagglutinin
HI: Hemagglutination inhibition
HP: Highly pathogenic
HPAIV: Highly pathogenic avian influenza virus
LP: Low pathogenic
M: Matrix
MAFFT: Multiple Alignment using Fast Fourier Transform
MCMC: Markov Chain Monte Carlo
NA: Neuraminidase
NDV: Newcastle Disease Virus
NEP: Nuclear export protein
NP: Nucleoprotein
NS: Non-structural
OIE: World Organisation for Animal Health
PA: Polymerase acidic
PB: Polymerase basic
RNA: Ribonucleic acid
RT-qPCRs: Real-time reverse-transcription polymerase chain reaction assays
SPF: Specific-pathogen-free
TRT: Turkey rhinotracheitis-
