# Supplementary material for: Isolation and genetic characterization of a novel 2.2.1.2a H5N1 virus from a vaccinated meat-turkeys flock in Egypt
Source: Virol J. 2017 Mar 9;14:48. doi: 10.1186/s12985-017-0697-5 (PMC5343302; doi:10.1186/s12985-017-0697-5)
Supplement: Additional file 1: Table S1. — Amino acids differences from A/turkey/Egypt/AR1507/2016 and ancestral 2006 virus, vaccine strain and closest human isolate (DOC 36 kb) [file 12985_2017_697_MOESM1_ESM.doc]

**Additional file 1: Table S1:** Amino acids differences from A/turkey/Egypt/AR1507/2016 and ancestral 2006 virus, vaccine strain and closest human isolate

| Segment | 2006 | Vaccine | Human |
| --- | --- | --- | --- |
| PB2 | M66I,K80R, T106A,T129N, K197R,E249K, I292M,M315I, R369K, I529V,M570I, A661T, D740N | V66I, V67I, K80R, S106A,N107S, A108T,T129N,V147T,K175R,K194Q,N195D,K197R,I292M,M315I,S327G,R368Q,R369K,D390N,L404F,S428L,S438F,I451T,Q508R,I511V,I529V,M570I,C590G,E627K,V649I,A661T**,**S711N,P728Q,D740N,F741S,N747S | D740N |
| PB1 | T182I, K214R, L384S, K387R | T57K, E104G, P162R, T182I, R206G, K214R, R215K,K352R, M363K, L384S, K387R, K390E, I644V, I709V, Q756P | K387R |
| PA | I94V, N321G, L342M, I348V, E351D,N359T, S388R, D394N,S400T,A448E, K615R,K626R, A669V, F707L, K716N | G58S,I94V,D101E,I129T,R204K,V231A,V261L,R269K,A287S, ↓319E,N321G,A337T,L342M,I38V,E351D,N359T,K367R,S388R,R391K,D394N,P400T,K401R,S404A,A448E,L486I,I554V,S560P,K615R,K626R,S631G,P653S,F707L,T712A,K716N | I348V, N359T,K367R,K372E,D394N,A669V |
| HA | L-4I,D43N,S120D, ∆129S,I151T,D154N,N155D,R162K,G272S,R325K,K373R,F537S | L-4I, D43N, I71L, A83I, S120D, ∆129S, K140R, P141S, I151T, N155D, T156A, R162K, I174V, S181P, K189R, D227E, P235S, Y252N, A263T, V269L, G272S, K310R, L322Q, ↓324E, E325K, R328K, K373R, K473R | V-7I,N-14K,G270E,K310R,D476N |
| NP | I33V,R98K,I109V,A129S,I186V,G287S, R452K | G34S,R98K,I109V,A129S,I363V,M371T,T373A,V408I,K446R, R452K | I343V, S432N |
| NA | V20A,M29I, V34I,A40T, A46D,P48S, T56A,I74V, R91K,N180S,L204M, V221I,V244I,T269I,V284, D378E, S430G | V20A,M29I,V34I,H44C,A46D,P48S,R50S,A52T,N53K,T56A,N58K,R75S,H80Y,N180S, L204M, V221I,N228S,V244I,N250D,T269I, V284I, S319F.I326V, E362G, N366S, D378E, R409G,S430G, | N46D,T269I |
| M1 | I15V, R95K ,I168T, N207S | I15V,I59M,R95K,I168T,N207S |  |
| M2 | C50F, T65M, N82S | V28I, C50F, T65M | T65M, N82S |
| NS1 | R44K, N48S, E55K E71K, E76A, A150T, N166S, I193V, P211S, T220A, K224E | R44K, N48S, E55K, E71K, T81A, A107T, F133Y, R149G, A150T, G166S, S172A, F180L, T192I, I193V, Y196F, S201I, L210P, P211S, N212D, T220A |  |
| NS2 | M9V | K10R, I19M, Q34R, G36E, T44S, V49L, F58L, V83I, A115T |  |

New mutations compared to the parent 2.2.1 virus which were not reported before are highlighted in green
