# Supplementary figures and images for: Isolation and genetic characterization of a novel 2.2.1.2a H5N1 virus from a vaccinated meat-turkeys flock in Egypt
Source: Virol J. 2017 Mar 9;14:48. doi: 10.1186/s12985-017-0697-5 (PMC5343302; doi:10.1186/s12985-017-0697-5)

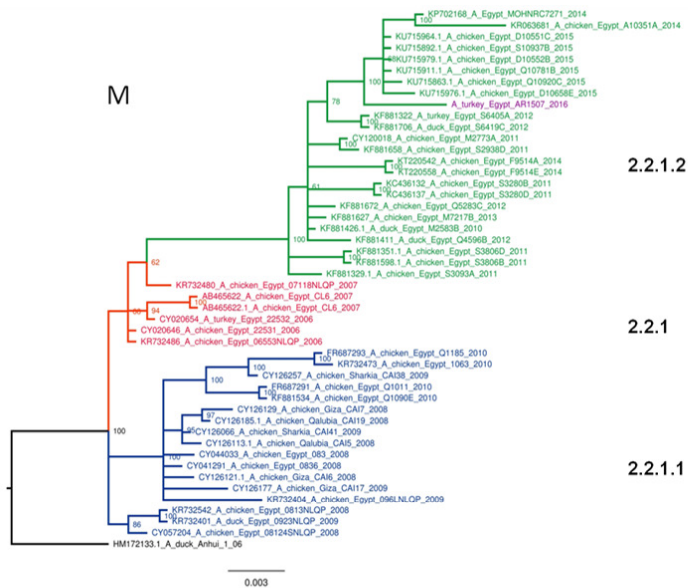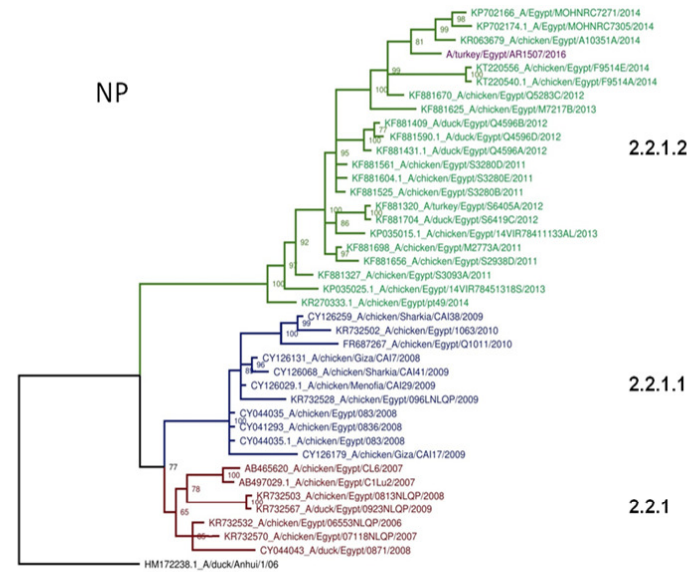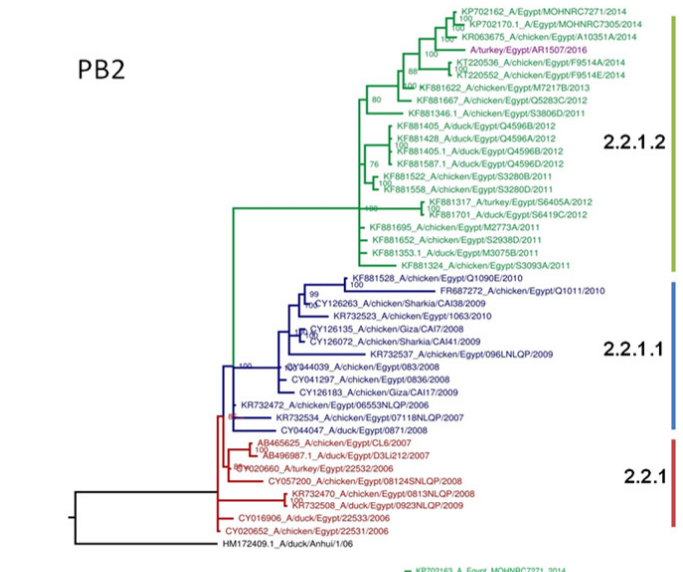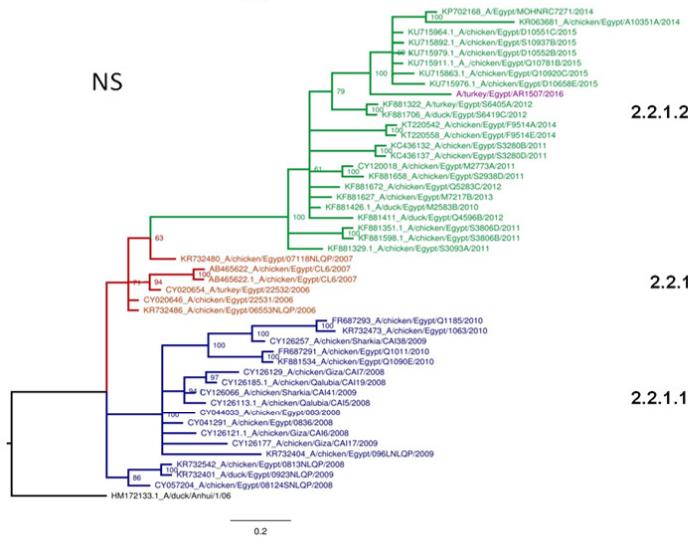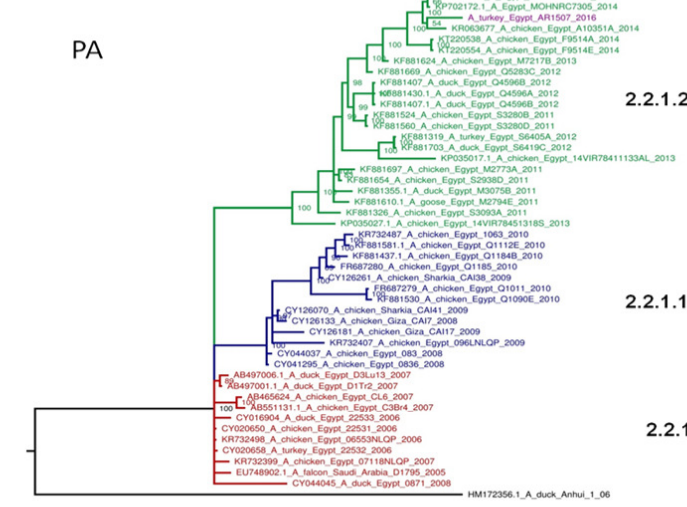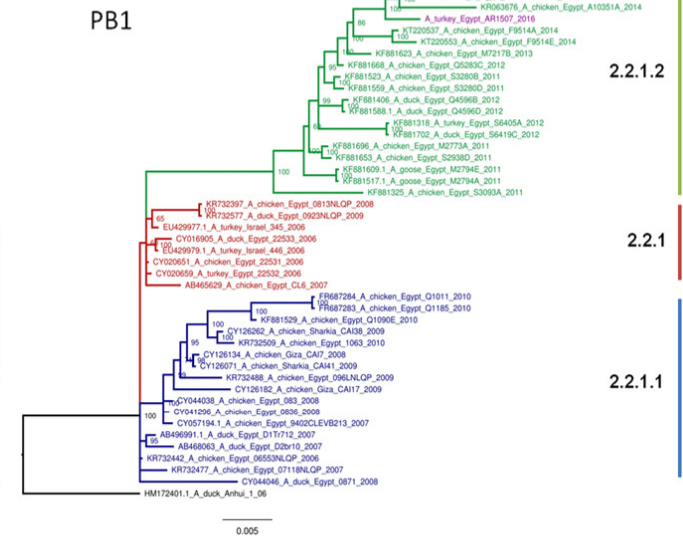

Supplement: Additional file 2: Figure S1. — Phylogenetic analysis of internal protein coding gene segments of A/turkey/Egypt/AR1507/2016 to other related H5N1 viruses in poultry and humans. Phylogenetic analysis of non HA/NA genes of Egyptian viruses showing different genetic clades. Viruses in 2015 from Egypt including A/turkey/Egypt/AR1507/2016 are clustered in clade 2.2.1.2. The tree was generated by MrBayes under the best-fit model selected by jModelTest. (PDF 701 kb) [file 12985_2017_697_MOESM2_ESM.pdf]
